# Retrospective SPECT/CT dosimetry following transarterial radioembolization

**DOI:** 10.1002/acm2.13213

**Published:** 2021-03-12

**Authors:** Briana C. Thompson, William A. Dezarn

**Affiliations:** ^1^ Department of Radiation Oncology Wake Forest Baptist Hospital Winston‐Salem NC USA; ^2^ Wake Forest School of Medicine, Molecular Medicine and Translational Sciences Winston Salem NC USA

**Keywords:** dose distribution, image‐based dosimetry, Radioembolization, Y90 Bremsstrahlung SPECT/CT

## Abstract

Transarterial radioembolization (TARE) effectively treats unresectable primary and metastatic liver tumors through intra‐arterial injection of Yttrium‐90 (^90^Y) beta particle emitting microspheres which implant around the tumor. Current dosimetry models are highly simplistic and there is a large need for an image‐based dosimetry post‐TARE, which would improve treatment safety and efficacy. Current post‐TARE imaging is ^90^Y bremsstrahlung SPECT/CT and we study the use of these images for dosimetry. Retrospective image review of ten patients having a Philips Healthcare^TM^ SPECT/CT following TARE SIR‐Spheres® implantation. Emission series with attenuation correction were resampled to 3 mm resolution and used to create image‐based dose distributions. Dose distributions and analysis were performed in MIM Software SurePlan^TM^ utilizing SurePlan^TM^ Local Deposition Method (LDM) and a dose convolution method (WFBH). We sought to implement a patient‐specific background subtraction prior to dose calculation to make these noisy bremsstrahlung SPECT images suitable for post‐TARE dosimetry. On average the percentage of mean background counts to maximum count in the image across all patients was 9.4 ± 4.9% (maximum = 7.6%, minimum = 2.3%). Absolute dose increased and profile line width decreased as background subtraction value increased. The average value of the LDM and WFBH dose methods was statistically the same. As background subtraction value increased, the DVH curves become unrealistic and distorted. Background subtraction on bremsstrahlung SPECT image has a large effect on post‐TARE dosimetry. The background contour defined provides a systematic estimate to the activity background that accounts for the scanner and patient conditions at the time of the image study and is easily implemented using commercially available software. Using the mean count in the background contour as a subtraction across the entire image gave the most realistic dose distributions. This methodology is independent of microsphere and software manufacturer allowing for use with any available products or tools.

## 1 Introduction

Transarterial radioembolization (TARE) uses beta particle emitting microspheres to treat unresectable primary and metastatic liver tumors. These Yttrium‐90 (^90^Y) containing spheres are injected intra‐arterially to surround the tumor(s), where they will become lodged in the tumor microvasculature delivering 95% of the radiation dose within 11 days due to its half‐life of 64.041 hours.^1^, ^2^ The mean range for radiation delivery is 2.5 mm, with a maximum penetration depth of 11 mm in tissue, allowing for local irradiation of tumor cells while sparing healthy liver tissue.^3^, ^4^ The efficacy of this treatment is made possible due to the liver having a dual blood supply. A healthy liver receives a vast majority of its blood supply via the portal vein and the remainder through the hepatic artery. In the case of primary and metastatic liver tumors, the tumor itself typically receives all of its blood supply through the hepatic artery.^5^ Therefore, an interventional radiologist is able to create a map of the patient’s vasculature to then determine a path to the tumor(s) site where they will then inject the microspheres. During this pretreatment planning, Technetium‐99m macroaggregated albumin particles (^99m^Tc‐MAA) are used to mimic the dispersion of microspheres upon treatment and are considered an acceptable surrogate for the basis of dosimetry calculations.^3^, ^6^, ^7^, ^8^ Although they act as an acceptable prediction for microsphere distribution there is still a need for post‐TARE emission imaging for validation of microsphere placement, especially for patient safety.^1^, ^9^, ^10^, ^11^, ^12^

Upon completion of TARE treatment, a ^90^Y bremsstrahlung single‐photon emission computed tomography (SPECT) imaging study is acquired to identify any extrahepatic microsphere deposition.^3^, ^4^, ^13^ Historically, these images are only used only for qualitative information due to their noisy nature but some groups have shown their potential in quantitative imaging upon extra image processing.^3^, ^14^, ^15^ Many challenges are present using these ^90^Y bremsstrahlung SPECT images quantitatively. One is due to the continuous bremsstrahlung spectrum with a wide photon energy range of 0‐2.3 MeV.^1^, ^16^ Another challenge is a lack of distinct photopeaks that are routinely used in SPECT imaging in addition to high‐energy photons being able to penetrate through the collimator septa.^1^, ^14^, ^16^ Just these few concerns lead to very noisy, low resolution, images.

There is a general consensus on the benefits of accurate image‐based post‐TARE dosimetry among researchers in the field but a lack of information on best practices using commercially available software makes implementation across intuitions difficult.^1^, ^3^, ^6^, ^17^ Recently there have been advancements in understanding the promising nature of post‐TARE ^90^Y positron emission tomography (PET) image‐based dosimetry, however, it needs to be noted that not all institutions may have the feasibility to obtain ^90^Y PET images post‐TARE. At such locations, it is still imperative to perform post‐TARE dosimetry thus there is still a need to better understand how to best use bremsstrahlung SPECT images for accurate dosimetry. In this study, we sought to create a way to address background noise on these post‐TARE SPECT images on an easily implemented patient‐specific basis and determine background subtraction effects on resulting dose distributions.

## 2 Methods

**Fig. 1:**
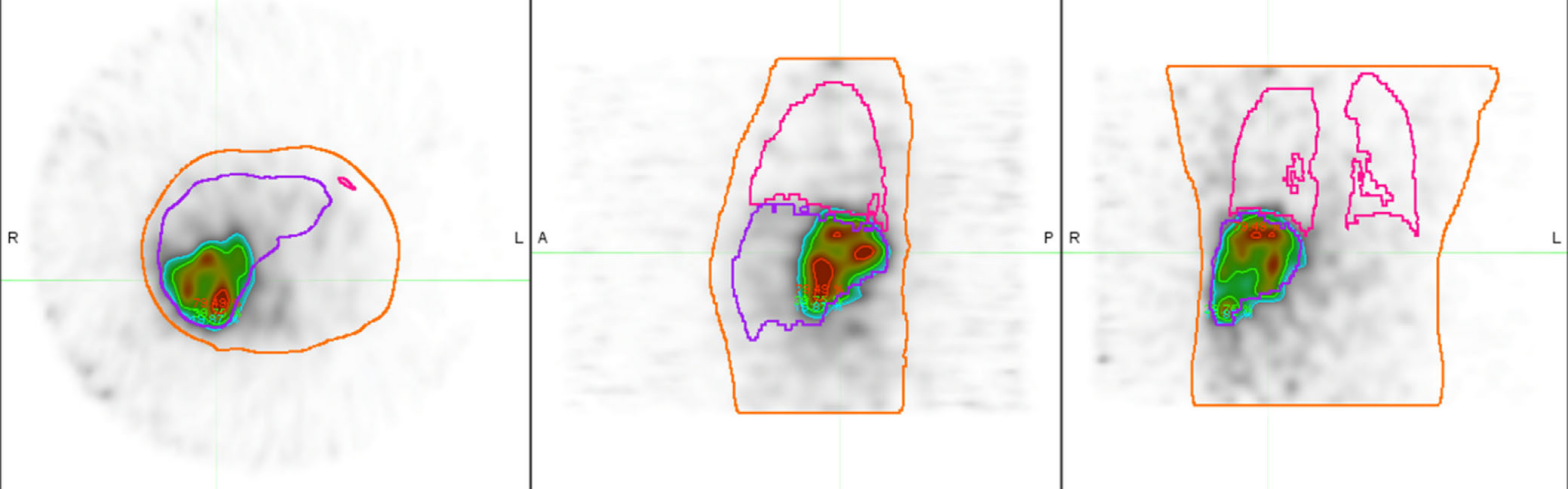
Dose distribution example. Representative SPECT image showing WFBH + Bkrgd dose method. The whole body is contoured in orange, liver in purple, and lungs in pink. Red denotes 100 Gy, green is 50 Gy and blue is 25 Gy. Note the noisy nature of the image.

Retrospective creation of post‐TARE image‐based dose distributions for ten patients treated with SirTex SirSpheres^TM 90^Y microspheres for hepatic malignancies at Wake Forest Baptist Medical Center. Each patient had a bremsstrahlung SPECT/CT (parameters shown in Table 1) utilizing a Philips Healthcare^TM^ scanner following SIR‐Spheres® implantation. This study has been approved by the institutional review board and the need for written informed consent was waived. The SPECT images were attenuation corrected with the CT data, which was the only scanner correction. Using MIM Software SurePlan^TM^ (MIM Software Inc., Cleveland, OH) the SPECT images were resampled to 3 mm resolution and contours were drawn for organs of interest based on the registered CT image. Dose distributions, as shown in Fig. 1, were created based on these bremsstrahlung SPECT images using SurePlan^TM^ Local Deposition Method with scaling (LDM) and our own dose convolution method (WFBH), both applied at the voxel level. Through the process of creating dose distributions, the emission image was scaled based on actual delivered activity at the time of treatment. Within the LDM model, the normalization and dose calculation is based on counts found within the liver contour. We expanded this contour by 20 mm on all sides prior to any normalization in order to account for potential movement during imaging, as well as if activity is located close to the edge of the liver. The expansion margin of 20 mm was chosen from our external beam radiotherapy experience with liver movement due to the patient’s breathing. For WFBH, the normalization is based on the total counts found in the body contour. Table 2 shows the patient characteristics and treatment details of those included for this analysis.

**Table 1:** Post‐TARE SPECT/CT imaging parameters.

Imaging parameter	Value
Energy window	328.04 keV – 400.94 keV
Collimator type	Parallel, medium energy
Postreconstruction filter	Hanning
Scan arc	360°
Number of frames in rotation	64
Type of detector motion	Step and collect
FOV dimensions	597 mm x 597 mm
Voxel size	4.664 mm x 4.664 mm x 4.664 mm

**Table 2:** Patient characteristics who were included in this study.

Patient no.	Sex	Lung Shunt	Treated Volume	Liver (cm^3^)	Tumor (cm^3^)	SMAC Activity (GBq)	Activity Delivered (GBq)
1	Male	4.39%	Rt Lobe	1715.5	129.51	0.97	1.04
2	Female	2.42%	Rt Lobe	2065.7	521.39	1.07	1.07
3	Female	3.32%	Seg 4	2109.5	73.37	0.25	0.25
4	Male	6.47%	Rt Lobe	1839.8	73.03	1.00	1.05
5	Female	1.10%	Rt Lobe	1880.3	478.46	1.37	1.39
6	Female	5.11%	Rt Lobe	1756.8	204.60	1.60	1.70
7	Male	3.59%	Rt Lobe	2212.9	304.23	0.97	1.00
8	Female	2.79%	Rt Lobe	1990.7	138.99	0.90	0.93
9	Female	2.41%	Rt Lobe	2741.2	690.66	1.25	1.14
10	Female	1.46%	Lt Lobe	1972.8	218.61	0.90	0.95

**Fig. 2:**
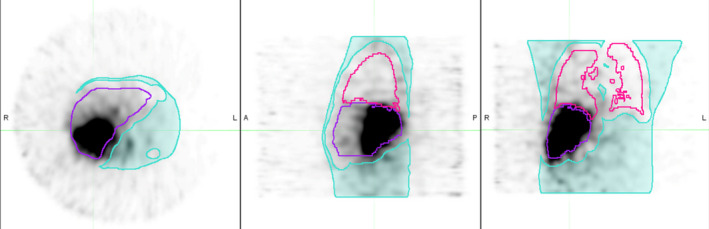
Background contour example. Representative SPECT image showing the background contour in blue, liver in purple, and lungs in pink.

The main goal of this study was to understand how the noise within these bremsstrahlung SPECT images affects the resultant image‐based dose distributions. In order to try get a handle on eliminating the background noise in the image while not eliminating true counts, we created what we will call a background contour. This background contour is defined to be the whole body contour minus the liver and lungs contours expanded each by 20 mm prior to subtraction. This contour was originally created on the CT image and transferred to the emission series where its statistics were recorded. Figure 2 shows an example of this contoured area, shaded in blue. In addition to dose distributions created using LDM and WFBH dose calculation methods on the original SPECT images, we created dose distributions after a constant background subtraction on the emission image was performed. We wrote an extension in Java (Oracle corporation et al., Redwood City, CA, USA) to run in MIM (see supplementary material for code) in order to easily subtract background on a patient by patient case. The extension prompts you to enter a background value (in counts) which will be subtracted from the entire emission image on a voxel basis. If a given voxel value becomes negative due to the subtraction, it will set to zero (0). We defined the following shorthand for the various corrected images we created based on the different background contour statistics being subtracted: Bkgrd = the mean counts found in background contour subtracted from all voxels, Bkgrd + SD= Bkgrd value plus one standard deviation subtracted, Bkgrd + 2SD = Bkgrd value plus two standard deviations subtracted. Table 3 shows a summary of the background contour statistics for the patients in this study. Once we performed all three background subtractions on the original emission image, we then created dose distributions using LDM and WFBH methods for each corrected image, thus in total, for each patient we had eight dose distributions. Four dose distributions were created using LDM, one from the resampled image and the three others from the background subtracted levels. Similarly, four dose distributions were created using WFBH method.

**Table 3:** Statistics for the background contour for each patient based on SPECT counts.

Patient no.	Mean background count	Background count max	Background count standard deviation
1	5.0	76.8	4.8
2	5.1	27.4	3.6
3	1.6	15.4	1.5
4	6.5	58.3	5.6
5	7.7	47.0	5.8
6	3.9	20.9	2.7
7	6.6	40.1	5.0
8	4.7	38.0	4.1
9	6.5	32.2	4.3
10	5.1	85.0	3.8

Dose volume histogram (DVH) curves and dose profiles were used to compare the different dose distributions obtained in this study. Dose profiles were created by defining the max dose point, obtained via WFBH Bkgrd method, and drawing a sagittal line passing through this point. This line was transferred to all dose images for consistency in the spatial dimension for the line profile comparisons.

## 3 Results

**Fig. 3:**
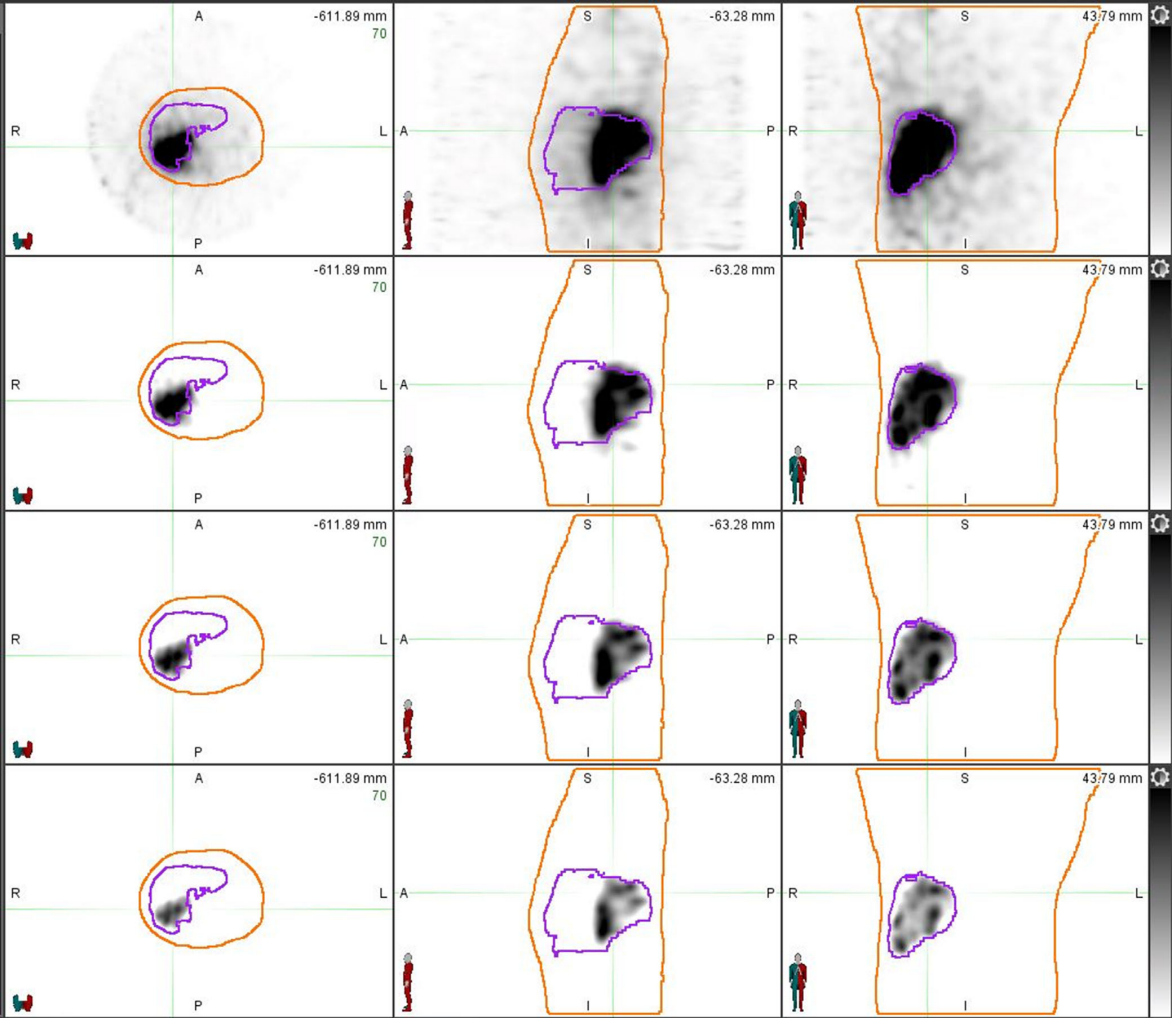
Visual representation of background subtraction on image. Representative images showing for the same patient, the effects of our background subtraction technique. Each row represents a different subtraction value starting from the top row having no subtraction, second row mean value subtraction, third being mean + SD and last row is mean + 2SD.

**Fig. 4:**
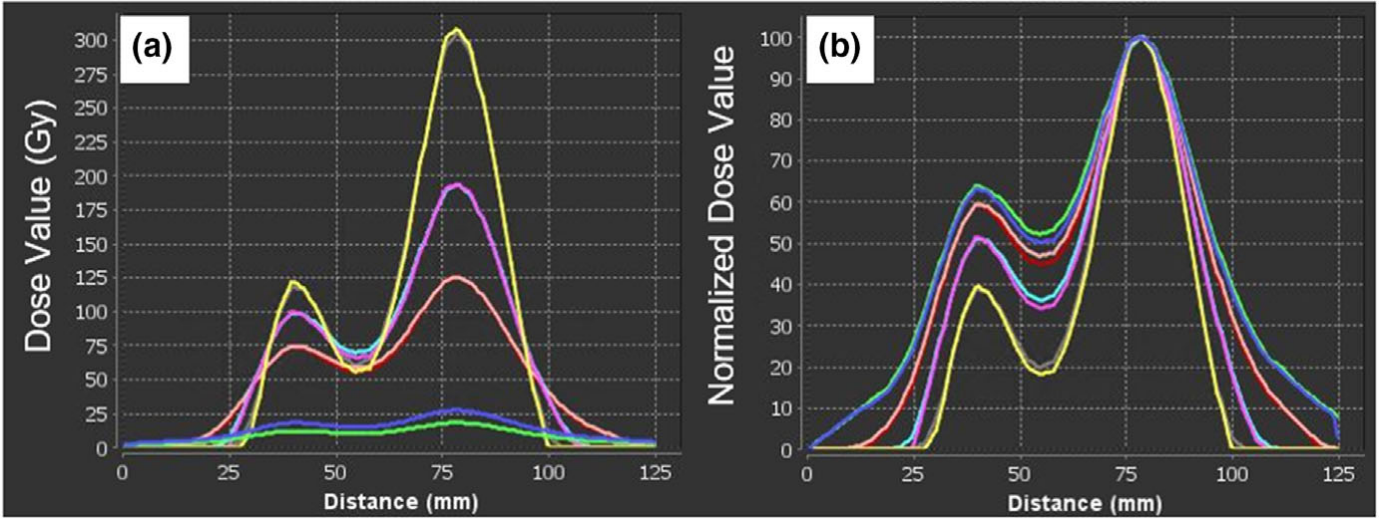
Representative dose profile. Sagittal dose profile through the max dose point. Fig. 4(a) shows the dose value in Gy on the y‐axis and an arbitrary location on the x‐axis. Fig. 4(b) shows the normalized dose value on the y‐axis and an arbitrary location on the x‐axis. Dark blue line represents WFBH method, green is LDM method, peach is WFBH Bkgrd, red is LDM Bkgrd, magenta is WFBH Bkgrd + SD, light blue is LDM Bkgrd + SD, yellow is WFBH Bkgrd + 2SD, and gray is LDM Bkgrd + 2SD.

Figure 3 demonstrates the visual effects of our background subtraction technique showing the effectiveness of our technique. However, the question then becomes at what point are true counts being artificially subtracted out of the image as we increase the background value. Table 3 allows us to better understand the magnitude of the background. The background values range from 9.4% ± 4.9 % to 23.5% ± 11.6% of the max count value in the image, showing we are subtracting a substantial count value compared to the largest value in the image. Additionally, Table 3 shows that although most counts within this background contour are small, you can see that there are still large values within this area, just furthering the point that this background subtraction is necessary considering that in theory, there should be no counts found within this contour. By subtracting these low count values uniformly across the image we are decreasing the amount of noise and low dose in the image. As expected, Table 4 shows that as the subtracted background value increases, the max dose value increases for both dose calculation methods. This trend further demonstrates that a statistically defined background subtraction is needed. No background correction can predict too low of a dose delivered while too high of a background subtraction will predict too high of a dose delivered. The dose line profiles in Figure 4a demonstrate that absolute dose is effected by background subtraction, the maximum dose increases as the background subtraction value increases. When the dose line profiles are normalized to the maximum point in each respective method, we see that dose distributions are not spatially being effected for a given background level (Figure 4b). Also note in Figure 4b the low dose region shrinking as the background subtraction value increases. This demonstrates the potential of removing low dose delivered regions that could be important to consider in future patient care. Through these dose profiles we see an agreement between LDM and WFBH dose calculation methods and Table 5 shows the agreement of the resulting maximum dose values. LDM yielded higher maximum dose values on average without background consideration but upon background subtraction the two methods give comparable ratios.

**Table 4:** Effect of background subtraction on maximum dose value.

	Ratio of max dose values between background subtracted images and original images					
	WFBH Bkgrd: WFBH	WFBH Bkgrd + SD: WFBH	WFBH Bkgrd + 2SD: WFBH	LDM Bkgrd: LDM	LDM Bkgrd + SD: LDM	LDM Bkgrd + 2SD: LDM
Average	6.692	10.503	15.864	4.805	7.516	11.270
Standard Deviation	2.351	4.056	7.483	2.012	3.439	6.005

**Table 5:** Comparison of LDM and WFBH maximum dose values averaged over all patients.

	Ratio of max dose values obtain via LDM and WFBH methods			
	LDM: WFBH	LDM Bkgrd: WFBH Bkgrd	LDM Bkgrd + SD: WFBH Bkgrd + SD	LDM Bkgrd + 2SD: WFBH Bkgrd + 2SD
Average	1.378	0.970	0.967	0.966
Standard Deviation	0.205	0.106	0.105	0.106

**Fig. 5:**
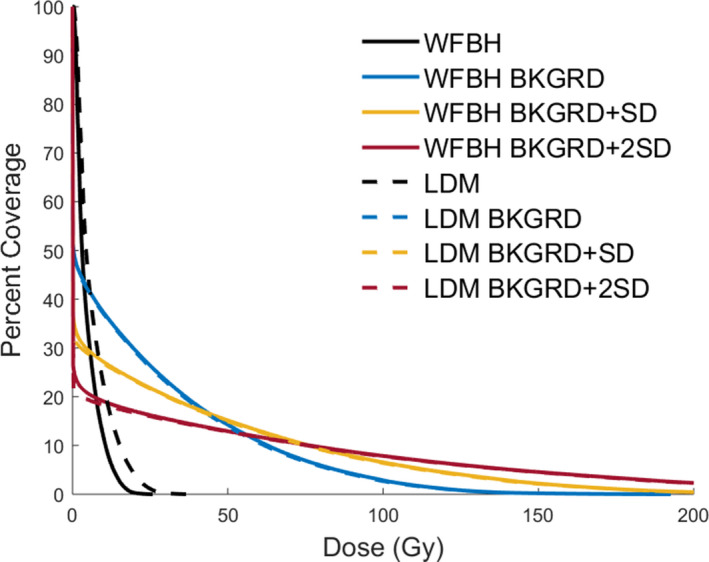
Liver DVH curves. Showing the effect background subtraction has on the liver DVH curves for both dose calculation methods. We see dose method has little effect while background subtraction value has large effect.

**Fig. 6:**
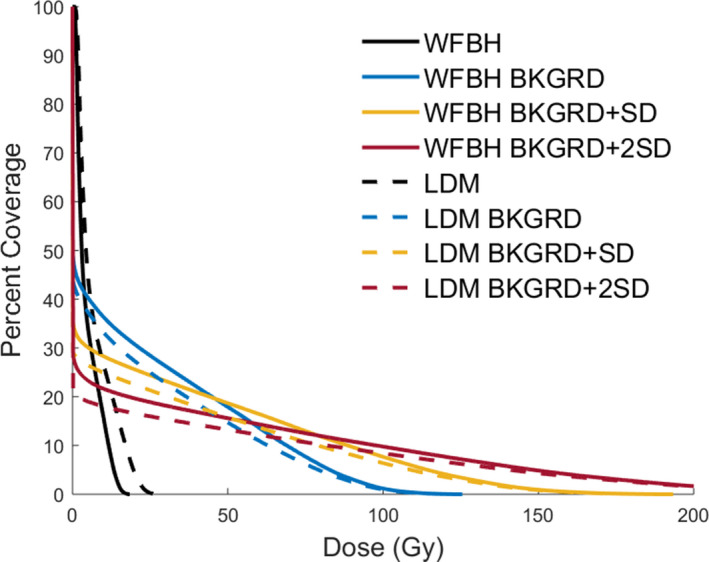
Example of statistically different DVH curves, We found that in a majority of cases these DVH curves between LDM and WFBH dose methods with and without background subtraction were not statistically different, however, this is an example of when this was not the case.

Figure 5 shows representative DVH curves for the listed liver dose distributions. We found that DVH curves for WFBH and LDM methods were not statistically different in 39 of 44 curves through two‐tailed p‐values (statistical significance deemed to be less than 0.05). In these five curves that were deemed statistically different, three were from the same patient and were for the resulting DVH curves after each background subtraction value. Figure 6 shows the DVH curves for this patient and as we see the LDM method is shifted below the WFBH method in these three cases. The other two statistically different curves were for just one set of curves in each patient. Across all three patients their treatment area is right at the edge of the liver contour.

**Fig. 7:**
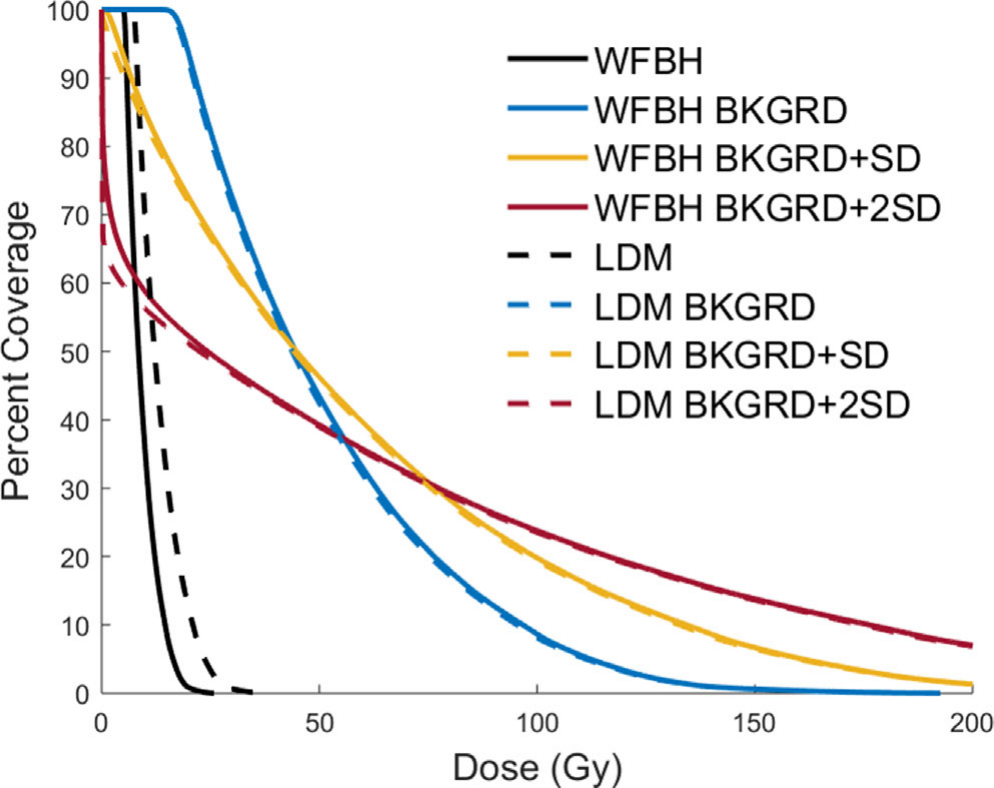
Treated region DVH curves. Both dose calculation methods are effected by background subtraction in similar manners. As we increase the background subtraction value we see unrealistic distortion of the treated region DVH curves.

In Figures 5, 6, 7 WFBH Bkgrd and LDM Bkgrd show an expected shape change for DVH curves due to the decrease in low dose and increase in high dose as well as healthy tissue sparing. Figure 7 shows DVH curves for the treated region, defined to be the region that is up to 20% of the max activity. These curves demonstrate better dose coverage with Bkgrd subtraction value compared to no correction. At the same time, Bkgrd + SD and Bkgrd + 2SD DVH curves show that these background values are unphysically skewing the delivered dose distribution to less coverage at low dose values than no background correction hence an unphysical altering of dose distribution at these levels. With Bkgrd subtraction level we do not see this effect and it is deemed the largest value we can subtract without creating unphysical changes in the DVH curves.

## 4 Discussion

Although ^90^Y PET imaging is thought to be superior for post‐TARE dosimetry due to improved spatial resolution,^18^, ^19^, ^20^ it cannot be assumed that all institutions performing these treatments have access to such imaging modalities. Therefore, a better understanding of the dosimetric outcomes based on bremsstrahlung SPECT images is still necessary. By utilizing commercially available software in this study we are also enhancing the ability for post‐TARE image‐based dosimetry calculations to become standard practice, something that is highly lacking in this field currently due to the imaging complexities associated with ^90^Y.

We explored one methodology of trying to eliminate false counts in the emission scan prior to dosimetry calculations. Theoretically, there should not be activity present within the background contour. SPECT images have activity within the background contour due to the natural background present in any radiation experiment. Sources of background come from processes such as: reconstruction artifacts, radiation sources outside of the patient near the imaging suite, and poor scanner performance, to name a few. We postulate that using a background contour for each image series to estimate the background correction is the most systematic method because it provides a statistical sample from the same data set that the dosimetry is being calculated. Other imaging corrections can be considered but only the image set itself demonstrates the background present in the imaging environment.

As the background value increased in magnitude, the overall max dose of the resulting dose distributions to increased, which was expected because absorbed dose within a voxel is calculated via the following equation:

$$D_{\mathrm{voxel}}=\frac{A\left(1‐LSF\right)T_{1/2}E_{\mathrm{avg}}C_{\mathrm{voxel}}}{\Delta V\rho \ln\left(2\right)C_{\mathrm{total}}}$$

where *A *=activity, *LSF* = lung shunt fraction, *T*_1/2_ = ^90^Y half‐life, *E_avg_* = average β‐particle energy per disintegration (0.935 MeV), *C_voxel_* = counts within voxel, Δ*V *= voxel volume, ρ = tissue density, and *C_total_* = total counts within the patient.^17^ The calculated dose in a given voxel is the voxel value divided by the total counts multiplied by a constant. So as the background subtraction level increases, the count total decreases, which is in the denominator, thus causing the calculated dose value to increase.

An assumption of the LDM model is that all the energy from β‐particle decay is locally deposited within the same voxel.^17^ WFBH method uses a 5x5x5 scattering kernel to consider the effects on neighboring voxels. However, through our analysis in this study it appears this does not make a considerable difference in resulting dose distributions when using ^90^Y bremsstrahlung SPECT images. This is likely due to the mean range of β‐particles in tissue being 2.5 mm and using a resampled voxel size of 3 mm in our emission scan.

Overall, either LDM or a convolution method like WFBH applied at the voxel level with the mean counts in the background contour subtracted uniformly across the image gives the most realistic post‐TARE dosimetry based on ^90^Y bremsstrahlung SPECT images. Further work needs to address how these models with mean background subtraction compare to dose distributions based on ^90^Y PET images to further validate accuracy.

## 5 Conclusion

Image processing, such as background subtraction, has a large effect on post‐TARE SPECT dosimetry. Both LDM and WFBH convolution method applied at the voxel level with constant background subtraction appear to give reasonable post‐TARE dosimetry based on DVH curves and dose profiles. We defined easy to implement steps for image based on ^90^Y bremsstrahlung SPECT post‐TARE images despite their inherent noisy nature. Our recommended protocol for post‐TARE image‐based dosimetry using bremsstrahlung SPECT can be found in Table 6. Our goal is to educate and emphasize the importance of post‐TARE image‐based dosimetry in order to move the field toward making background subtracted SPECT images a standard protocol in order to improve patient treatment dosimetry and outcomes.

**Table 6:** Protocol recommendations for post‐TARE SPECT‐based dosimetry.

1	Resample this background subtracted SPECT image to 3 mm resolution
2	Contour the whole body, liver and lungs on CT
3	Expand liver and lung contours by 20 mm to account for organ motion from breathing
4	Subtract the union of expanded liver and lung contour from whole body
5	Transfer contour to resampled SPECT image
6	Record the mean counts within this new background contour
7	Run background subtraction extension (code found in supplemental information) by selecting the SPECT image, input mean background count value and save new background subtracted image
8	Use a voxel‐based dosimetry method on 3 mm resampled background subtracted SPECT image

## Author contribution

Both authors contributed to the conception, design, and analysis of the study. Data collection was performed by BCT. First draft of the manuscript was written by BCT and WAD commented and edited on each version of the manuscript. Both authors read and approved the final manuscript and assume accountability for all aspects of the work.

## Conflict of Interest

BCT declares no conflict of or competing interests. WAD serves as an advisory board consultant for Sirtex Medical.

## Supporting information

Supplementary Material
