# Supplementary material for: Retrospective SPECT/CT dosimetry following transarterial radioembolization
Source: J Appl Clin Med Phys. 2021 Mar 12;22(4):143–50. doi: 10.1002/acm2.13213 (PMC8035553; doi:10.1002/acm2.13213)
Supplement: Supplementary file 1 — Supplementary Material [file ACM2-22-143-s001.pdf]

```java
package sample.control;

import com.mimvista.external.control.XMimEntryPoint;
import com.mimvista.external.control.XMimSession;
import com.mimvista.external.data.XMimMutableNDArray;
import com.mimvista.external.points.XMimNoxelPointI;
import com.mimvista.external.series.XMimImage;
import com.mimvista.external.series.XMimMutableImage;
import com.mimvista.external.series.XMimSeriesView;

import javax.swing.JOptionPane;

/**
 * @author dezarnwa
 *
 *  Main entry point for EmissionTomographyBackground MIM extension
 */
public class EmissionTomographyBackground {

    private static final String desc = "Subtract constant background values from emission image.";

    /*
     *  @XMimEntryPoint tells MIM that this function can be used to start a MIMextension
     *       Meta-data entered here will display in the extension launcher inside MIM
     */
    @XMimEntryPoint(
            name="Emission Tomography Background",
            author="William A Dezarn",
            category="Utility",
            description=desc,
            institution="WFBH",
            version="1.0",
            outputTypes={XMimImage.class})
    public static Object[] runOnSession(XMimSession session, XMimImage image) {
        // Run the code
        XMimImage result = process(session, image);

```

```
        //Once we finish processing the result, add it to the session so it can be displayed
        XMimSeriesView view = session.addImageAndReturnView(result, "Bkgd Subtracted");

        //Our @XMimEntryPoint says we return one XMimImage object, so do that here
        //This allows our MIMextension to integrate with workflows
        return new XMimSeriesView[] {view};
    }

    public static XMimImage process(XMimSession session, XMimImage image) {
        if(image.getRawData().getDimensionCount() < 3) {
            session.createLogger().error("Insufficient dimensions; requires a volumetric image");
            return null;
        }

        // MIMextensions don't allow a volume to be modified in place
        // So, create a mutable copy so we can edit it
        XMimMutableImage mutable = image.getMutableCopy();
        // getScaledData() Provides the scaled data array of this image. Want BQML for PET, so use scaled.
        XMimMutableNDArray CountData = mutable.getScaledData();
        int[] dimensions = CountData.getDims();


        // Ask for background
        String EntryString = JOptionPane.showInputDialog("Enter background level.");
        Double Bkgd = Double.parseDouble(EntryString);

        // Following section subtracts background from image counts or sets to zero if less than background
        XMimNoxelPointI point = image.createNoxelPointI(); // voxel coordinates used to get data
        // Loop over entire image to subtract background and initialize dose array
        for(int z=0; z<dimensions[2]; z++) {
            point.setCoord(2, z);
```

```
            for(int y=0; y<dimensions[1]; y++) {
                point.setCoord(1, y);
                for(int x=0; x<dimensions[0]; x++) {
                    point.setCoord(0, x);

                    Double value = 1.0 * CountData.getDoubleValue(point); // get image counts
                    if (value <= 1.0*Bkgd) {                        // subtract background as needed
                        CountData.setValue(point, 0.0);
                    } else {
                        CountData.setValue(point, (value-1.0*Bkgd));
                    }

                }
            }
        }

        //JOptionPane.showMessageDialog(null, "Activity map created. TotCount = " + TotCount.toString());

        return mutable;
    }
}
```
